# Development of Real-Time and Conventional PCR Assays for Identifying a Newly Named Species of Root-Lesion Nematode (*Pratylenchus dakotaensis*) on Soybean

**DOI:** 10.3390/ijms22115872

**Published:** 2021-05-30

**Authors:** Intiaz Amin Chowdhury, Guiping Yan

**Affiliations:** Department of Plant Pathology, North Dakota State University, Fargo, ND 58108, USA; intiaz.chowdhury@ndsu.edu

**Keywords:** detection, DNA, endomigratory, identification, in silico analysis, ITS rDNA, conventional PCR, *Pratylenchus dakotaensis*, real-time PCR, root-lesion nematode

## Abstract

A rapid and accurate PCR-based method was developed in this study for detecting and identifying a new species of root-lesion nematode (*Pratylenchus dakotaensis*) recently discovered in a soybean field in North Dakota, USA. Species-specific primers, targeting the internal transcribed spacer region of ribosomal DNA, were designed to be used in both conventional and quantitative real-time PCR assays for identification of *P. dakotaensis*. The specificity of the primers was evaluated in silico analysis and laboratory PCR experiments. Results showed that only *P. dakotaensis* DNA was exclusively amplified in conventional and real-time PCR assays but none of the DNA from other control species were amplified. Detection sensitivity analysis revealed that the conventional PCR was able to detect an equivalent to 1/8 of the DNA of a single nematode whereas real-time PCR detected an equivalent to 1/32 of the DNA of a single nematode. According to the generated standard curve the amplification efficiency of the primers in real-time PCR was 94% with a R^2^ value of 0.95 between quantification cycle number and log number of *P. dakotaensis*. To validate the assays to distinguish *P. dakotaensis* from other *Pratylenchus* spp. commonly detected in North Dakota soybean fields, 20 soil samples collected from seven counties were tested. The PCR assays amplified the DNA of *P. dakotaensis* and discriminated it from other *Pratylenchus* spp. present in North Dakota soybean fields. This is the first report of a species-specific and rapid PCR detection method suitable for use in diagnostic and research laboratories for the detection of *P. dakotaensis*.

## 1. Introduction

Root-lesion nematodes, *Pratylenchus* spp., are one of the most economically important nematode pests of crops worldwide [1]. These plant-parasitic nematodes have a migratory endoparasitic nature, ability to reproduce sexually and/or asexually, excellent adaptability to diverse environmental conditions, and broad host range [2]. Thus, they are ranked as the third most important group of plant-parasitic nematodes after root-knot nematodes and cyst nematodes [3]. Furthermore, there are over 70 described species of *Pratylenchus* affecting crops of major economic importance [4], including cereals, coffee, fruits, ornamental crops, peanuts, soybeans, and vegetables, resulting in serious economic losses for growers throughout the world [2,4].

Numerous studies have reported high yield losses caused by root-lesion nematodes in infested crop fields. In Australia, *P. thornei* was reported to cause yield losses in wheat as high as 85% [5]. In Oregon USA, *P. neglectus* reduced the yield of intolerant wheat cultivars by 36% [6]. In Norway, *P. penetrans* caused tuber lesions and reduced potato yield by 50% in affected fields [7]. In Brazil, *P. brachyurus* was reported to cause 21% yield reduction in soybean fields [8]. Hence, appropriate and effective management of these nematode diseases is critical for improving crop production worldwide. The most commonly used management strategies against *Pratylenchus* spp. include crop rotation and host resistance [9]. Choice of crops available for rotation depends on the identity of the species of root-lesion nematode present in the field because the host range of each *Pratylenchus* sp. can vary and only a few plant species and cultivars are known to be immune or possess some level of resistance against these plant-parasitic nematodes [2].

The species identity of root-lesion nematodes is often determined using traditional microscopic methods, which utilizes subtle morphological differences such as vulval position, stylet length, body length, postuterine sac length, and lip annule number [2,10]. However, such methods are highly time-consuming because it requires experienced nematologists to collect detailed microscopic measurements from multiple nematode specimens from each field to generate morphometric values [2,10]. Furthermore, multiple species of *Pratylenchus* can coexist in a single field, making it more challenging to identify *Pratylenchus* spp. [11,12,13]. On the other hand, molecular technologies provide several rapid, reliable, and efficient DNA-based diagnostic methods for the species identification of root-lesion nematodes.

One of the first such molecular diagnostic studies on root-lesion nematodes was described by Pinochet et al. [14] that identified and differentiated isolates of *P. vulnus* using random amplified polymorphic DNA (RAPD) polymerase chain reaction (PCR). Since then, numerous studies have identified and differentiated *Pratylenchus* spp. to the species level using DNA-based molecular diagnostic methods such as PCR restriction fragment length polymorphism (RFLP), species-specific PCR, and DNA sequencing [15,16,17,18,19,20,21,22]. The RFLP potion of the RFLP–PCR is an additional time-consuming step that is not amenable to commercial high-throughput applications [22]. Although recent advances in sequencing technology simplified the sequencing process, it can still be relatively expensive when a large number of samples need to be diagnosed. Species-specific PCR being a simple, rapid, and relatively inexpensive method, is commonly used in nematology research for diagnostic purposes [3,17].

Conventional species-specific PCR method uses PCR amplification with species-specific primers followed by gel electrophoresis to identify the species of the target organism based on bands visible in agarose gel. These species-specific primers are often designed based on sequence information of ribosomal DNA (rDNA) such as the D2–D3 expansion region of 28S rDNA and internal transcribed spacer (ITS) of rDNA [2,17]. Additionally, specific genes such as cytochrome c oxidase gene, β-1,4-endoglucanase gene, and major sperm protein gene are commonly used to design species-specific primers in plant-nematology [2].

Al-Banna et al. [15] developed conventional PCR assays with five forward species-specific primers, PNEG, PSCR, PPEN, PTHO, and PVUL, to identify and differentiate *P. neglectus*, *P. scribneri*, *P. penetrans*, *P. thornei*, and *P. vulnus*, respectively. Each of the five forward primers and a single common reverse primer were designed from the D3 expansion region of 28S rDNA. However, the difference in amplicon size between some of the target species was as little as two base-pairs. Thus, Yan et al. [22] modified and designed the PNEG-F1/D3B5 primer set, which could specifically identify *P. neglectus* by conventional PCR. Additionally, Yan et al. [22] optimized the amplification conditions for the PTHO/D3B primer set to successfully identify *P. thornei*. Mekete et al. [23] designed the PP5 primer set from ITS rDNA region to identify *P. penetrans* using conventional PCR and multiplex PCR assays. Huang and Yan [17] designed the PSF7/PSR7 primer set from the ITS-rDNA region to identify *P. scribneri*. Moreover, Huang and Yan [17] reported that the PSF7/PSR7 primer set was equally effective in identifying *P. scribneri* using conventional and real-time PCR. Compared to conventional PCR, real-time PCR is a molecular diagnostic technique that has higher detection sensitivity, provides quantitative data, and allows real-time data monitoring [24]. Many other studies have also developed species-specific primers for detection of root-lesion nematode species such as *P. brachyurus*, *P. coffeae*, *P. crenatus*, *P. loosi*, *P. neglectus*, *P. penetrans*, *P. scribneri*, *P. thornei*, and *P. zeae* using species-specific PCR or real-time PCR [16,19,20,21,25,26,27].

In North Dakota, during 2015 and 2016, soil surveys of soybean fields were conducted to determine the prevalence and distribution of plant-parasitic nematodes in these fields [28]. During the surveys, six soil samples were collected from a soybean field in Walcott, Richland County, ND. After extracting nematodes from these samples, it was evident that root-lesion nematodes were present in all of these samples with population densities ranging from 125 to 2000 nematodes per kg of soil. Morphological measurement of adult males and females and DNA sequencing of 28S D2-D3 (GenBank accession: KX889989) and ITS rDNA (KX889990) revealed that this nematode differs from other known species of root-lesion nematodes in both morphology and DNA sequences [28]. Greenhouse experiments proved that this *Pratylenchus* species reproduced well on soybeans with a reproductive factor (final population/initial population) as high as 5.02, thus, allowing us to conclude that this is a new species of *Pratylenchus* that had never been reported in the literature prior to 2017. Recently, Handoo et al. [29] named this new species of root-lesion nematodes as *Pratylenchus dakotaensis*, paying homage to the state in which it was discovered. However, no species-specific PCR diagnostic method exists to identify this nematode species prior to this study. In order to detect and distinguish *P. dakotaensis* from other *Pratylenchus* spp., new species-specific primers need to be exploited for the development of an efficient and sensitive detection system.

The objectives of this research were to develop conventional and real-time PCR assays with species-specific primers for rapid, reliable, and sensitive detection of *P. dakotaensis*, and to evaluate the capability of the assays to discriminate between *P. dakotaensis* and other commonly found *Pratylenchus* spp. in North Dakota soybean fields.

## 2. Results

### 2.1. Species-Specific PCR Primer Design

The multiple sequence alignment of the ITS rDNA from *Pratylenchus* spp. revealed a unique region in the genome of *P. dakotaensis* that is polymorphic between different species. The species-specific primer set, IC-ITS1F/IC-ITS1R designed based on the region was expected to amplify a specific 194-bp fragment of the ITS rDNA of *P. dakotaensis*. The percent GC content for the 18-bp forward primer (IC-ITS1F) was 50%, whereas the percent GC content for the 22-bp reverse primer (IC-ITS1R) was 41%. The predicted annealing temperatures for IC-ITS1F and IC-ITS1R were estimated as 57.6 and 58.9 °C, respectively. Additionally, the primers had a low potential for duplex formation through hairpin formation, self-dimerization, and heterodimer formation. Using the BLAST search function of the nr/nt database of NCBI for the initial specificity screening revealed that the primer sequences have no perfect match with non-target plant-parasitic or non-plant-parasitic nematodes and no strong similarity to other soil microorganisms.

The IC-ITS1F and IC-ITS1R primers were predicted to have strong and specific annealing with the ITS rDNA sequence of *P. dakotaensis* based on primer-template duplex stability (ΔG) values of the in silico analysis (Table 1). Since ΔG values less than −31 kcal/mol predict stable primer-template hybrid formation, the IC-ITS1F and IC-ITS1R primers, having a ΔG value of −33.5 and −38.4, respectively, were predicted to hybridize with the ITS rDNA sequence of *P. dakotaensis* [20,30]. Values of ΔG higher than 31 kcal/mol are indicative of unstable primer-template binding resulting in unidirectional or non-logarithmic amplification, which are not competitive with specific amplification. Thus, IC-ITS1F forward primer was predicted to hybridize poorly with ITS rDNA sequences of the three *P. neglectus* populations used in silico analysis, having a ΔG value of −14.4. On the other hand, the IC-ITS1R reverse primer was predicted to form insignificant (ΔG = ins) hybrid with ITS rDNA sequences of *P. neglectus* [20,30]. In addition to that, both the primers were not predicted to result in primer-template duplexes with the ITS rDNA sequences of any other *Pratylenchus* spp. used in silico analysis (Table 1). Hence, the primer set IC-ITS1F/IC-ITS1R was predicted to specifically amplify the DNA of *P. dakotaensis*. Therefore, this primer set was selected for further laboratory experiments.

### 2.2. Primer Specificity

**Figure 1:**
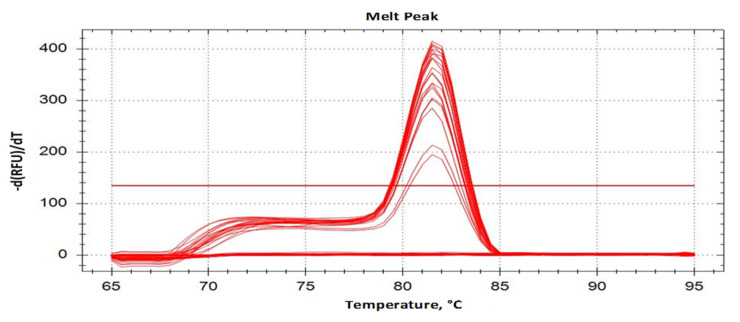
Melting curve profiles of *P. dakotaensis*. Amplicons with a single peak at a melting temperature of 81.5 °C were observed for populations of *P. dakotaensis*.

Laboratory conventional and real-time PCR assays confirmed the prediction of in silico analysis about the specificity of the newly designed primers. As expected, the IC-ITS1F/IC-ITS1R primer set amplified a specific 194-bp DNA fragment of the target species of *Pratylenchus* (*P. dakotaensis*). A single specific amplicon was observed in the conventional PCR assay with all four populations *P. dakotaensis* used in the specificity tests (Table 2, sample ID: Hg50-1, Hg50-2, Hg50-3, and Hg50-4). Additionally, all DNA extracted from different numbers of the target individuals (4, 2, 1, and 0.5) from the same population produced the specific amplicon in conventional PCR (Table 2, Hg50-4). No amplification was observed in the conventional PCR assay with non-target control species consisting of multiple populations of five other confirmed *Pratylenchus* spp., eight other genera of plant-parasitic nematodes, and two genera of non-plant-parasitic nematodes. Conventional PCR also amplified DNA from each of the target and non-target species using the universal primers rDNA2/rDNA1.58s, confirming the presence of template DNA in each of the samples tested. As expected, real-time PCR amplifications with the non-target control species yielded no fluorescence signal, thus Cq values were not detectable. However, Cq values ranging from 26.43 ± 0.05 to 30.64 ± 0.50 were recorded from real-time PCR with the various populations of *P. dakotaensis* at varying numbers of *P. dakotaensis* individuals in DNA extracts (Table 2). Melting curve analysis revealed a single melting peak at 81.5 °C and no secondary curves confirming a single amplicon specific for each of the four populations of *P. dakotaensis* (Figure 1). For both conventional and real-time PCR assays, no amplicons were generated in non-template controls containing water instead of DNA.

### 2.3. Detection Sensitivity of the PCR Assays

**Figure 2:**
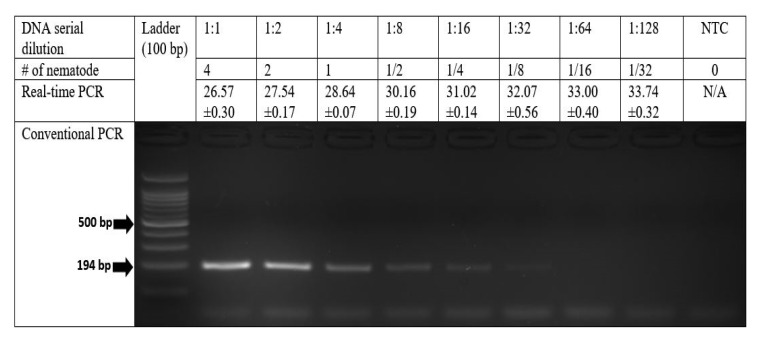
Amplification sensitivity of the IC-ITS1F/IC-ITS1R primer set in conventional polymerase chain reaction (PCR) compared to real-time PCR. The quantification cycle (Cq) value is presented as the mean ± standard deviation of three technical replicates of one of the biological replicates used for standard curve development. DNA was extracted from 4 *P. dakotaensis* individuals and sequential two-fold serial dilutions (2, 1, 1/2, 1/4, 1/8, 1/16, 1/32, and 1/64) were conducted, but the last dilution level 1:256 (1/64) was not included in this figure.

**Figure 3:**
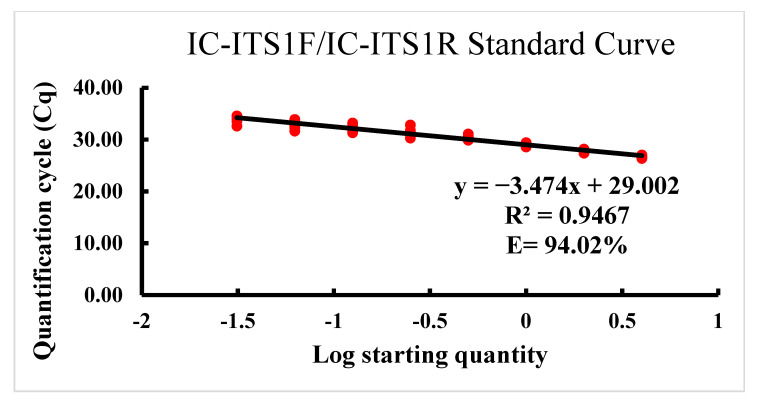
Standard curve of the real-time PCR assay for the new species of *Pratylenchus* detected in a soybean field of North Dakota: quantification cycle number (Cq) plotted against the log number of individuals of *P. dakotaensis* by sequential two-fold dilutions (4, 2, 1, 1/2, 1/4, 1/8, 1/16, 1/32, and 1/64). Each red dot represents an independent reaction run in triplicate for three biological replicates at each dilution level. The efficiency (E) of amplification was calculated as E = 1^1/-*m*^ − 1, where *m* indicates the slope.

The sensitivity was determined using DNA from each dilution level of a sequential two-fold serial dilution of DNA extracted from four *P. dakotaensis* individuals in both conventional and real-time PCR assays. As expected, with each dilution of template DNA the intensity of PCR bands gradually decreased in the conventional PCR assay, and the Cq values were continuously increased in the real-time PCR assay (Figure 2). According to gel electrophoresis of conventional PCR products on 2% agarose gel, the assay was able to amplify the DNA of four *P. dakotaensis* individuals diluted down five times (4, 2, 1, 1/2, 1/4, and 1/8) to an equivalent of 1/8 of a single nematode. However, DNA from 6th (1/16), 7th (1/32), and 8th (1/64) dilutions were not amplified by the conventional PCR indicated by the absence of bands on 2% agarose gel, suggesting that the IC-ITS1F/IC-ITS1R primer set could detect an equivalent to 1/8 of the DNA of a single *P. dakotaensis* individual. In the real-time PCR assay, DNA from each dilution level was used to generate a standard curve. The equation for the standard curve was determined as y = −3.474x + 29.002, with an R^2^ value of 0.9467 and an efficiency (E) of 94.02%. Thus, the standard curve generated showed a strong negative correlation between the Cq values from real-time PCR and the log values of numbers of *P. dakotaensis* (Figure 3). Since the amplification efficiency and the R^2^ value were high and within the acceptable range, the IC-ITS1F/IC-ITS1R primer set was considered suitable for real-time PCR assay. The sensitivity of the real-time PCR assay was also determined. The assay was able to amplify the DNA of four individuals diluted seven times (4, 2, 1, 1/2, 1/4, 1/8, 1/16, and 1/32), with the Cq values ranging from 26.57 ± 0.30 to 33.74 ± 0.32 (Figure 2) and the DNA from the 8th dilution (1/64) was not amplified, suggesting this primer set could detect an equivalent to 1/32 of the DNA of a single nematode in the real-time PCR assay.

### 2.4. Identification of Pratylenchus *spp.* Collected from Soybean Fields

The 20 soil samples collected from soybean fields or fields with a history of soybean production across seven soybean producing counties of North Dakota (Cass, Dickey, Grand Forks, Nelson, Richland, Sargent, and Wells) were all positive with *Pratylenchus* spp. The population densities of *Pratylenchus* spp. in these field soil samples ranged from 15 to 360 nematodes per 100 cm^3^ of soil (Table 3). Out of the 20 samples, 12 were determined to be *P. neglectus* whereas seven were determined to be *P. scribneri* through published species-specific PCR assays. The IC-ITS1F/IC-ITS1R primer set designed in this study did not amplify any of those 19 samples, however it was able to amplify the DNA of root-lesion nematodes obtained from a soil sample (Table 3, sample ID: 50 RL 3) that was collected from a field that is neighboring the field where *P. dakotaensis* was first detected and from a soil sample collected from the original field (HG50-0). Subsequent direct sequencing of D2-D3 expansion region of 28S rDNA and ITS rDNA region validated that 50 RL 3 was infested with *P. dakotaensis*.

## 3. Discussion

*Pratylenchus* spp. are one of the most economically important pests of field crops [3,33]. However, the potential of root-lesion nematodes to cause economic loss can vary according to the species present and their population density [34]. Therefore, diagnostic assays that can effectively identify the species of *Pratylenchus* are of the utmost importance to facilitate their management. Conventional and real-time PCR continues to be amongst the most important diagnostic tools in plant nematology research [3], since they are relatively inexpensive, less time consuming, accurate, and easy to use.

Both conventional and real-time PCR have advantages of their own, compared to each other. While conventional PCR is more inexpensive, easy to use, and less affected by PCR inhibitors [35], real-time PCR is less time consuming, more sensitive, allows continuous monitoring of data, and provides quantitative data [20]. Thus, we designed and developed a conventional PCR assay, and a SYBR Green-I based real-time PCR assay, for identification and quantitative detection of *P. dakotaensis*, which is a new species of *Pratylenchus* recently discovered in a soybean field in North Dakota [28,29]. The IC-ITS1F/IC-ITS1R primer set designed was able to specifically and sensitively amplify the DNA of *P. dakotaensis* in both conventional and real-time PCR assays. Moreover, the designed primer set was able to successfully detect and differentiate the *P. dakotaensis* from other confirmed *Pratylenchus* spp. collected from 20 other fields of North Dakota. Hence, we report for the first time, two efficient molecular methods for detection and identification of a new species of *Pratylenchus* (*P. dakotaensis*) detected in North Dakota [28].

The IC-ITS1F/IC-ITS1R species-specific primer set was designed from the ITS rDNA region of *P. dakotaensis*’ genome. Numerous ITS-region based PCR assays have been developed to identify and differentiate plant-parasitic nematodes to their species level [17,25,30,36,37,38,39,40,41]. Compared to 28S rDNA and 18S rDNA that are conserved through evolution, the ITS rDNA region is considered to be more variable among genera and species of plant-parasitic nematodes and other soil-borne organisms [17,42,43]. Although, several studies have developed species-specific primers that were designed from the 18S rDNA and 28S rDNA [15,16,22], the ITS rDNA sequences are known to have greater variability between different *Pratylenchus* spp. [17,19,42]. Several other nematode genes such as mitochondrial cytochrome oxidase 1, mitochondrial cytochrome oxidase 2, 16D10 effector gene, and chorismate mutase effector gene have been used, in recent studies, to design species-specific primers for identification of nematode species [44,45,46,47,48]. However, in our literature review we found that more ITS rDNA sequences of plant-parasitic nematodes and *Pratylenchus* spp. were available in GenBank and previous literature compared to these genes, which facilitated our sequence analysis and comparison when designing the primers.

The performance of species-specific primers in PCR is substantially influenced by primer length, melting temperature (*T_m_*), GC content, and potential for self-dimer or primer dimer formation [49]. The specificity of a primer pair is generally controlled by their sequence length and the annealing temperature used in PCR reaction. Primers that are 18–24 bp in length are considered to be the best in being sequence specific if the annealing temperature of the PCR reaction is set within 5 °C of the primer *T_m_* [49,50]. On the other hand, primers with 50–60% GC content ensure optimal binding strength of primer-template duplex because the G and C bases have stronger hydrogen bonding, and the binding strength of the primer-template duplex determines the efficiency of annealing [51]. Moreover, the presence of G or C bases at the 5’ and 3’ end ensures a GC clamp and promotes specific binding. Another important factor to consider when designing a primer is the potential of the primers to form secondary structures. Presence of complementary sequence within the primer length can result in hairpin or self-dimer formation, whereas homology between the forward and reverse primer can lead to primer dimer formation [52]. These secondary structures can directly compete with the template in PCR, negatively affecting species-specific amplification [50]. Thus, the IC-ITS1F and IC-ITS1R primers were designed to have optimal primer length, melting temperature (*T_m_*), GC content, and low potential for secondary structure formation.

Numerous previous molecular diagnostic studies have used in silico analysis to predict the performance of primers regarding specificity against multiple populations of their respective target species and non-target species [20,21,22,25,30,53,54,55]. The in silico analysis provided us an opportunity to predict the specificity of the primers against the DNA sequences of multiple populations of 26 other important species of *Pratylenchus* originating from different regions of the world. Results of in silico analysis predicted that the primers were highly specific to *P. dakotaensis*. The specificity of the primers was further confirmed through laboratory PCR assays with multiple populations of *P. dakotaensis* and multiple populations of five other *Pratylenchus* spp. along with non-target control species in eight other genera. Since DNA from each of the target nematode population were amplified by the designed primers and none of the non-target species’ DNA were amplified, the results of the PCR specificity tests were consistent with the predictions obtained from the in silico analysis. This indicates that the IC-ITSF/IC-ITS1R primer set can be successfully used in both conventional and real-time PCR assays to distinguish and identify *P. dakotaensis* from other plant-parasitic nematode species evaluated. Nevertheless, *Pratylenchus* is a diverse genus containing more than 70 species (4). Further testing of these primers with other *Pratylenchus* spp. and local species in soil may be necessary if these primers were used for diagnosis in other geographic regions.

Primers that are specific for other *Pratylenchus* spp. in conventional PCR have been described before [2,15,22]. However, not all of those primers could directly be used in real-time PCR due to non-specific amplifications being detected by the greater amplification sensitivity of real-time PCR [17,21]. However, the species-specific primers designed in this study can be used in both conventional PCR and real-time PCR for species identification. Applicability of the primers in real-time PCR was demonstrated by the high inverse linear relationship observed between the Cq values and the log values of nematode numbers (R^2^ = 0.95) revealed by the standard curve generated. Moreover, the standard curve also revealed that the primers had good amplification efficiency (E = 94%), reflecting on the robustness of real-time PCR assay developed.

Practically, DNA from as little as a single nematode could be used to specifically identify *P. dakotaensis* using the molecular diagnostic methods developed in this study. As expected, the real-time PCR assay with greater sensitivity could detect an equivalent of 1/32 of a single nematode; whereas, the conventional PCR could detect an equivalent of 1/8 of a single nematode. These results are in agreement with findings from previous studies that developed species-specific primers can be used in both conventional and real-time PCR assays for nematode identification [17,30]. Moreover, the sensitivity of the conventional and real-time PCR assays was comparable to other nematode related PCR assays reported in previous literature, where DNA from less than a single nematode individual could be detected [17,20,26,30,36,56,57]. For examples, the real-time PCR assay developed by Berry et al. [26] could detect as little as 2.5% (1/40) of the DNA of a *P. zeae* individual and the conventional PCR assay developed by Huang and Yan [17] could detect an equivalent of 1/4 of the DNA of a single *P. scribneri* individual.

The assays developed in this study were able to discriminate between *P. dakotaensis* and other *Pratylenchus* spp. commonly present in North Dakota soybean fields. Among the 20 other fields of ND, from which soil samples were collected, a majority were infested with *P. scribneri* or *P. neglectus*. Although, several studies reported that multiple species of *Pratylenchus* could coexist in an individual cropping field [11,12,13], none of the fields surveyed in this study were found to be infested with more than one species of *Pratylenchus* by testing three nematode individuals from each field. The IC-ITS1F/IC-ITS1R primer set did not amplify the DNA of *P. scribneri* or *P. neglectus* obtained from these field samples. However, it was able to amplify the DNA of a *Pratylenchus* sp. obtained from a soil sample (Table 3, sample ID: 50 RL 3) collected from a neighboring field of the soybean field that was originally infested with *P. dakotaensis* [28]. Therefore, this study reports for the first time that another soybean field (50 RL 3) of North Dakota is infested with the new species *P. dakotaensis*.

In conclusion, the PCR assays developed in this study provides a rapid, specific, and sensitive detection method for *P. dakotaensis*. Both the specificity and sensitivity of the primers designed were confirmed through extensive testing. Moreover, the standard curve analysis revealed that the primers had a good amplification efficiency. Thus, the IC-ITS1F/IC-ITS1R primer set is suitable for use in both diagnostic laboratories and research laboratories for the detection of field infestation with this nematode species. The PCR-based methods can be performed on a single nematode, which overcomes the need of morphometric measurements of multiple, decent nematode specimens for species identification, and also can accurately determine species identity without the influence of variations in nematode morphology among different populations of the same species. Therefore, the information presented in this study would be valuable for improving nematode detection to develop nematode management strategies. Moreover, this study will help in the future development of PCR assays that can identify and quantify this nematode species directly from DNA extracts of soil or plant samples. This will simplify the diagnosis process by avoiding the nematodes extraction from soil or plant roots prior to DNA extraction.

## 4. Materials and Methods

### 4.1. Soil Sampling and Nematode Extraction from Soil

Soil samples were collected from a soybean field in Walcott, Richland County, ND, which was previously reported to have been infested with *P. dakotaensis* [28]. Nematodes were then extracted from the soil samples using the standard hand sieving, decanting, and sugar centrifugal–floatation method [58]. Briefly, this method relies principally on agitating the soil in tap water (200 mL) through stirring and pouring the mixture through a stack of sieves (250 µm and 20 µm apertures) to separate larger organic and inorganic soil particles from nematodes and smaller soil particles. Then, the smaller soil particles and nematodes that were collected in the 20-µm-aperture sieve were separated from each other by suspending them in a 1.3 M sucrose solution (American Crystal Sugar Company, Moorhead, MN, USA). Once suspended, the solution was centrifuged at 4000 RPM for 30 s. The supernatant containing the vermiform nematodes was poured through 20-µm-aperture sieve to remove the sugar solution and the nematodes were then rinsed with tap water and collected in 15 mL tap water for quantification, and the pellet containing the soil particles was discarded. Subsequently, root-lesion nematodes were identified and enumerated at the genus level based on their key morphological features [32] under a dissecting microscope (Zeiss Stemi 305; Zeiss, Thornwood, NY, USA) at 80× magnification.

### 4.2. Root-Lesion Nematode Isolation and DNA Extraction

Individuals of the *Pratylenchus* spp. identified to the genus level were isolated from the nematode suspension, and the proteinase K method described by Kumari and Subbotin [31] was then used to extract DNA from the nematodes. According to this method, individual nematodes were hand-picked using a dental pick, placed into a concave slide containing 10 µL of sterilized double-distilled water, and cut into two pieces using the dental pick under the dissecting microscope. The 10 µL suspension containing the nematode pieces was then pipetted into a 0.5 mL sterile centrifuge tube containing 10 µL of extraction buffer (2 µL of 10X PCR buffer, 2 µL of proteinase K (µg/mL), and 6 µL double-distilled water). The tubes containing the extraction buffer and the nematode suspension were then vortexed and frozen at −20 °C for at least 30 min. Subsequently, the tubes were incubated at 65 °C in a water bath for 1 h, followed by incubation at 95 °C for 10 min to inactivate the proteinase K [31].

### 4.3. Direct Sequencing for Identity Confirmation

To confirm the species identity of the root-lesion nematodes extracted from samples that were collected from the field where *P. dakotaensis* was first discovered [28,29], two genomic regions were sequenced. The universal primers D2A (5′-ACAAGTACCGTGAGGGAAAGTTG-3′) and D3B (5′-TCGGAAGGAACCAGCTACTA-3′) were used to amplify the D2-D3 expansion region of 28S rDNA, following the PCR protocol described by Subbotin et al. [59]. Additionally, the universal primers rDNA2-V (5′-TTGATTACGTCCCTGCCCTTT-3′) and PnSeqR (5′-TTTCACTCGCCGTTACTAAGG-3′) were used to amplify the ITS rDNA region, as described by Vrain et al. [60]. The PCR products were then purified using the PCR Extract Mini Kit (5 PRIME INC., Gaithersburg, MD, USA) and shipped to Eurofins Genomics (Louisville, KY, USA) for direct sequencing. The sequencing results were evaluated using the BioEdit software version 7.0.9.0 [61] and a Blastn alignment was performed in National Center for Biotechnology Information (NCBI, https://blast.ncbi.nlm.nih.gov/Blast.cgi, accessed on 4 April 2021) using the nucleotide collection (nr/nt) database to confirm the species identity by comparing to the sequences available in GenBank.

### 4.4. Species-Specific PCR Primer Design and Development

The internal transcribed spacer (ITS) rDNA sequence of *P. dakotaensis* (accession number KX889989) was acquired from GenBank [28]. Additionally, 16 ITS rDNA sequences from 11 other *Pratylenchus* spp. were collected from GenBank. These ITS sequences with the corresponding accession numbers contained two populations of each of *P. agilis* (FJ712891.1 and KC952982.1), *P. neglectus* (LC030328.1 and LC030323.1), *P. pseudocoffeae* (LC030337.1 and LC030339.1), *P. scribneri* (KT873860.1 and KX842626.1), and *P. thornei* (FR692305.1 and FR692304.1) and one population of each of *P. alleni* (JX081545.2), *P. gutierrezi* (KT971367.1), *P. hippeastri* (KR029085.1), *P. jaehni* (FJ712937.1), *P. loosi* (KY424222.1), and *P. penetrans* (LC030336.1). The ITS sequence of *P. dakotaensis* together with the ITS sequences of the 11 other *Pratylenchus* spp. were aligned using the Clustal W tool of the BioEdit software version 7.0.9.0 [61]. The multiple sequence alignment was used to identify and design a pair of primers, IC-ITS1F (forward, 5′-TGTGTGCGAATGTTCCTG-3′) and IC-ITS1R (reverse, 5′-CGTATGTTTTATATGGGGACTC-3′), within the diverse region of ITS rDNA, among the different species of *Pratylenchus* and the size of the target amplicon was 194 base pairs. The primer set was then assessed on the basis of optimal annealing temperature, GC content, and potential for duplex formation through hairpin formation, self-dimerization, and heterodimer formation using OligoAnalyzer 3.1 (Integrated DNA Technologies, Inc, Croalville, IA, USA).

The primers were initially screened for specificity using the BLAST search function in NCBI to determine if the primers matched with other non-target nucleotide sequences in the nucleotide collection (nr/nt) database. Moreover, the specificity of the primer set was predicted in silico against the 16 ITS rDNA sequences used to design the primers in addition to the ITS sequences of 16 other important *Pratylenchus* spp. collected from GenBank (Table 1). Primer specificity was determined in silico by evaluating the primer-template duplex stability values (ΔG) calculated using the OligoAnalyzer 3.1, as described by Schroeder et al. [55] and Yan et al. [20]. The primers were synthesized by Eurofin MWG Operon LLC (Huntsville, AL, USA). The performance of the designed primer set in PCR amplification was then evaluated at different annealing temperatures (56, 58, and 60 °C) using DNA from *P. dakotaensis*, along with DNA from *P. neglectus*, *P. penetrans*, *P. scribneri*, and *P. thornei*. Subsequently, 58 °C was determined to be the optimal annealing temperature at which the primer pair performed the best.

### 4.5. Detection Specificity of the Primers in PCR

The designed primer set was further evaluated for detection specificity through conventional and real-time PCR using DNA from multiple populations of the target species (*P. dakotaensis*) and multiple populations of five other confirmed species of *Pratylenchus* (Table 2). They included two populations of *P. scribneri* and *P. neglectus,* and one population of *P. thornei*, *P. penetrans*, and another newly identified *Pratylenchus* sp. (Table 2, sample ID: Hg 51). Additionally, DNA from eight populations of plant-parasitic nematodes in other genera, including *Paratylenchus* sp., *Paratrichodorus* sp., *Tylenchorhynchus* sp., *Helicotylenchus* sp., *Heterodera glycines*, *H. schatii*, *Hoplolaimus* sp., and *Xiphinema* sp., were used in the specificity test. Furthermore, DNA from two genera of non-plant-parasitic nematodes were also used in the specificity test (Table 2, sample ID: NPN1 and NPN2). For each nematode population used in the specificity testing, three independent DNA extractions were conducted and used as biological replicates.

### 4.6. Conventional PCR Assay

The presence and quality of the template in DNA extracts used in this study were verified using conventional PCR amplification of the ITS1 rDNA with the universal primer set rDNA2/rDNA1.58s [62]. The PCR amplification conditions were initial denaturing at 94 °C for 3 min followed by 40 cycles of denaturing at 94 °C for 45 s, annealing at 55 °C for 1 min, and extension at 72 °C for 1 min; with a final extension at 72 °C for 10 min, as suggested by Cherry et al. [62]. The Bio-Rad T100 thermal cycler (Hercules, CA, USA) was used to conduct all conventional PCR amplification reactions for this study. For the newly designed primer set, IC-ITS1F/IC-ITS1R, the species-specific conventional PCR reaction mixture (20 µL) consisted of 1.5 µL of DNA template, 0.5 µM forward and reverse primers, 0.2 mM dNTP, 1.5 mM MgCl_2_, 1X Green GoTaq Flexi buffer, 1 unit of GoTaq Flexi DNA polymerase (Promega Corp., Madison. WI, USA), and 11.2 µL of double-distilled water. The amplification conditions for the designed primer set were initial denaturation at 94 °C for 3 min followed by 35 cycles of denaturing at 94 °C for 40 s, annealing at 58 °C for 50 s, extension at 72 °C for 1 min, and a final extension for 10 min at 72 °C. After DNA amplification, 8 µL of the PCR products were added to the wells of a 2% agarose gel stained with ethidium bromide. The PCR products in the gel were then separated by gel electrophoresis for 30 min at 90 V. The banding pattern of the PCR products was visualized under UV light and photographed using the AlphaImager Gel Documentation system (Proteinsimple Inc., Santa Clara, CA, USA). For each sample, conventional PCR reactions were conducted with three biological replicates.

### 4.7. Real-Time PCR Assay

To determine whether the IC-ITS1F/IC-ITS1R primer set can be used in real-time PCR assay for the identification of *P. dakotaensis*, a SYBR-based real-time PCR assay was conducted. In the real-time PCR assay, a 12 µL qPCR reaction mixture contained 1.5 µL of DNA template, 6 µL of SsoAdvanced Universal SYBR Green Supermix (Bio-Rad), 0.5 µL of each forward and reverse primer (10 µM), and 3.5 µL of nuclease-free PCR grade water. In each experiment, non-template controls containing nuclease-free PCR grade water in place of DNA were included. The real-time PCR amplification was performed using the Bio-Rad CFX96 Touch Real-time PCR detection system (Bio-Rad Laboratories, Inc., Hercules, CA, USA.). The cycling conditions for the real-time PCR amplification were incubation at 95 °C for 4 min followed by 35 cycles of 95 °C for 10 s and 58 °C for 30 s. A melting curve temperature profile was generated by increasing the temperature of the reaction from 65 to 95 °C with a ramp rate of 0.5 °C s^−1^ to evaluate the amplification specificity. Each reaction was run in triplicate as technical replicates and three independent biological replicates were assessed for each sample, thus generating nine observations per sample in real-time PCR. The Bio-Rad CFX Manager Software (V3.1) was used to analyze the real-time PCR data, and the quantification cycle value (Cq) for each reaction was determined at default settings. The real-time PCR product quality was also evaluated by electrophoresis on a 2% agarose gel as described above.

### 4.8. Detection Sensitivity of PCR Assays

The sensitivity of the assays was investigated to establish a detection limit for the conventional and real-time PCR assays. The sensitivity, which refers to the minimum number of target nematode individuals that could produce visible amplicons in the gel electrophoresis portion of the conventional PCR assay or generate a Cq value lower than 34 in the real-time PCR assay [17,30], was evaluated using a sequential two-fold serial dilution of DNA extracted from four individuals of *P. dakotaensis*. Thus, DNA extracted from four *P. dakotaensis* individuals were diluted nine times down to 1/64 equivalent of a single nematode (4, 2, 1, 1/2, 1/4, 1/8, 1/16, 1/32, and 1/64). In a real-time PCR assay, each sequential dilution from three independent DNA extracts was then used as biological replicates and the PCR assay was conducted in triplicate as technical replicates. This resulted in nine observations for each dilution level in real-time PCR. The Cq values from each dilution level were then plotted against the corresponding log 10 of equivalent numbers of *P. dakotaensis* individuals to generate a standard curve, from which the coefficient of determination (R^2^) was determined and amplification efficiency was calculated using the equation, E = 10^(1/-m)^ − 1, where m is the slope of the equation. The same DNA templates from the sequential serial dilution of the first biological replicate were used to determine the detection limit for the conventional PCR assay.

### 4.9. Isolation, Identification, and Verification of Root-Lesion Nematode Species Collected from Soybean Fields

During 2019, 20 soil samples were collected from North Dakota soybean fields, or fields with a history of soybean production that were suspected to be infested with *Pratylenchus* spp. based on our previous research. One of these 20 soil samples was collected from a soybean field neighboring the field in which *P. dakotaensis* was first discovered. Additionally, in 2019, a soil sample was also collected from the field in which *P. dakotaensis* was first discovered. Nematodes from these samples were extracted and used to test whether the PCR assays could accurately detect and differentiate *P. dakotaensis* from other species of *Pratylenchus* (Table 3). In each field, sampling was conducted in a zig-zag pattern starting 76 m (100 paces) away from the field entrance, by collecting soil from 20–25 sampling points using a 2.5 cm diameter soil probe from depths of 1–30 cm below the soil surface [63]. After compositing and thoroughly mixing the soil samples collected from each field, nematodes were extracted from a 100 cm^3^ subsample using the sugar centrifugal–floatation method [58], as described previously. Nematodes belonging to the genus *Pratylenchus* were identified and enumerated based on morphological features using a compound microscope (100× magnification) and the density of *Pratylenchus* sp. in the 100 cm^3^ soil subsample was calculated. Individuals of *Pratylenchus* sp. were then isolated from each sample using a dental pick, and DNA was extracted using the Proteinase K method [31], as described previously. For each sample, single nematode DNA extractions were conducted independently from three different individuals of *Pratylenchus* as three biological replicates. The presence of DNA was confirmed using PCR with the universal primer set rDNA2/rDNA1.58s, as described by Cherry et al. [62]. Conventional species-specific PCR was then conducted to identify the *Pratylenchus* sp. in each sample. The primers used in species-specific PCR included PsF7/PsR7 [17], PNEG-F1/D3B5 [22], PTHO/D3B [22], and PP5F/PP5R [23], which are specific to *P. scribneri*, *P. neglectus*, *P. thornei*, and *P. penetrans*, respectively. The amplification conditions for the species-specific PCR followed the corresponding authors’ recommendations. Moreover, each DNA sample was tested in the conventional and real-time PCR assays with the newly designed primer set IC-ITS1F/ITS1R to confirm the specificity of the new PCR assays. The banding pattern of the PCR products in the conventional PCR assay and the Cq values from the real-time PCR assay were then recorded. Samples that were identified as *P. dakotaensis* through PCR assays with the IC-ITS1F/IC-ITS1R primer set were further validated through direct DNA sequencing by following the procedure described in Section 4.3.

## Figures and Tables

**Table 1:** Populations of *Pratylenchus* spp. and their corresponding GenBank accession numbers of internal transcribed spacer (ITS) sequences used for analysis of primer-template duplex stability (ΔG) for predicting the specificity of primers, IC-ITS1F and IC-ITS1R.

Species	GenBank Accession	Sample ID or Clone ID ^a^	Origin	Sequence Length (bp)	ΔG (kcal/mole) ^b^	
					IC-ITS1F	IC-ITS1R
*P. dakotaensis* ^c^	KX889990.1	Hg50	ND, USA	1126	−33.5	−38.4
*Pratylenchus* sp. ^d^	KY200666.1	Hg51	ND, USA	981	ins	ins
*P. agilis*	KC952982.1	SX/Clone7	P.R. China	882	ins	ins
*P. agilis*	FJ712891.1	PagKL5/Clone5	Belgium	880	ins	ins
*P. alleni*	JX081545.2	N/A	Canada	1080	ins	ins
*P. bolivianus*	HM469446.1	TW2	P.R. China	1163	ins	ins
*P. brachyurus*	MH020807.1	AD70	Spain	627	ins	ins
*P. coffeae*	JX046940.1	YT/Clone4	P.R. China	1102	ins	ins
*P. convallariae*	HM469448.1	N/A	P.R. China	722	ins	ins
*P. crenatus*	LC030310.1	He1/Clone1	Japan	928	ins	ins
*P. crenatus*	LC030308.1	Pcr-H01/Clone1	Japan	958	ins	ins
*P. fallax*	KY828273.1	V4C/Clone180	Belgium	755	ins	ins
*P. goodeyi*	KF700243.1	CICR-Cot.Warud	India	782	ins	ins
*P. gutierrezi*	KT971367.1	O22_1	Spain	906	ins	ins
*P. hippeastri*	KR029085.1	QIXIA	China	1185	ins	ins
*P. jaehni*	FJ712937.1	PjaKL1/Clone	Belgium	997	ins	ins
*P. lentis*	AM933154.1	Individual Nematode 10	Italy	703	ins	ins
*P. loosi*	KY424222.1	EX11	P.R. China	1212	ins	ins
*P. mediterraneus*	FR692306.1	N/A	Italy	946	ins	ins
*P. neglectus*	LC030328.1	NM1/Clone8	Japan	852	−14.4	ins
*P. neglectus*	LC030323.1	HT2KU1/Clone1	Japan	871	−14.4	ins
*P. neglectus*	LC030325.1	HKf1/Clone6	Japan	855	−14.4	ins
*P. penetrans*	LC030336.1	HM1/Clone3	Japan	874	ins	ins
*P. pinguicaudatus*	KY828261.1	T572/Clone168	Belgium	762	ins	ins
*P. pratensis*	KY828311.1	T616/Clone 232	Belgium	807	ins	ins
*P. pseudocoffeae*	LC030337.1	Pps-KM1/Clone1	Japan	1090	ins	ins
*P. pseudocoffeae*	LC030339.1	Pps-KM1/Clone7	Japan	1090	ins	ins
*P. scribneri*	KT873860.1	ND	ND, USA	1103	ins	ins
*P. scribneri*	KX842626.1	F21	ND, USA	1103	ins	ins
*P. scribneri*	JX046934.1	XC/Clone2	P.R. China	957	ins	ins
*P. thornei*	FR692305.1	Pt_Je_SP_cl13	Italy	1001	ins	ins
*P. thornei*	FR692304.1	PT_SI_IT_cl19	Italy	974	ins	ins
*P. vulnus*	KY424232.1	JSR	China	925	ins	ins
*P. zeae*	FJ643590.1	N/A	Republic of China	967	ins	ins

^a^ Names of samples or clones were obtained from GenBank. N/A indicates that the information is not available in GenBank. ^b^ Values of ΔG < −31 kcal/mol indicate stable primer-template duplex formation for PCR amplification, conversely, values > −31 kcal/mol indicate poor primer-template duplex stability for PCR amplification; ‘ins’ indicate very poor or insignificant primer-template duplex stability values. ^c^ The Hg50 nematode sample is the target species of *Pratylenchus* (*P. dakotaensis*) investigated in this study. ^d^ The Hg51 nematode sample is another species of *Pratylenchus* newly identified in North Dakota (ND), USA, which is different from *P. dakotaensis*.

**Table 2:** Taxon, geographic origin, current crop, and polymerase chain reaction (PCR) assays of the control nematode species used to test the specificity of IC-ITSF/ITS1R primer set designed to identify and detect *P. dakotaensis*.

Sample ID ^a^	Species	Origin	Crop ^b^	# of Nema ^c^	PCR Assay	
					Conventional ^d^	RT (Cq) ^e^
Ps1	*Pratylenchus scribneri*	Sargent, ND, USA	Corn	2	−	N/A
Ps2	*P. scribneri*	Sargent, ND, USA	Potato	2	−	N/A
Pn1	*P. neglectus*	Richland, ND, USA	Corn	2	−	N/A
Pn2	*P. neglectus*	Bottineau, ND, USA	Field Pea	2	−	N/A
Pt	*P. thornei*	OR, USA	Wheat	2	−	N/A
Pp	*P. penetrans*	Sherburne, MN, USA	Potato	2	−	N/A
Tyl	*Tylenchorhychus* sp.	Richland, ND, USA	Corn	2	−	N/A
Spi	*Helicotylenchus* sp.	Richland, ND, USA	Soybean	2	−	N/A
Xph	*Xiphinema* sp.	Sargent, ND, USA	Potato	2	−	N/A
Prt	*Paratylenchus* sp.	McIntosh, ND, USA	Corn	2	−	N/A
Ptr	*Paratrichodorus* sp.	Sargent, ND, USA	Potato	2	−	N/A
Hop	*Hoplolaimus* sp.	Sargent, ND, USA	Soybean	2	−	N/A
SCN	*Heterodera glycines*	Richland, ND, USA	Soybean	2	−	N/A
SBCN	*H. schachtii*	Richland, MT, USA	Sugarbeet	2	−	N/A
NPN1	Non-plant parasitic nematode 1	Richland, ND, USA	Corn	2	−	N/A
NPN2	Non-plant parasitic nematode 2	Richland, ND, USA	Soybean	2	−	N/A
Hg51	*Pratylenchus* sp.	Richland, ND, USA	Soybean	2	−	N/A
Hg50-1	*P. dakotaensis*	Richland, ND, USA	Soybean	2	+	28.91 ± 0.40
Hg50-2	*P. dakotaensis*	Richland, ND, USA	Soybean	2	+	27.70 ± 0.10
Hg50-3	*P. dakotaensis*	Richland, ND, USA	Corn	2	+	28.02 ± 0.59
Hg50-4	*P. dakotaensis*	Richland, ND, USA	Soybean	4	+	26.43 ± 0.05
Hg50-4	*P. dakotaensis*	Richland, ND, USA	Soybean	2	+	27.60 ± 0.20
Hg50-4	*P. dakotaensis*	Richland, ND, USA	Soybean	1	+	29.87 ± 0.50
Hg50-4	*P. dakotaensis*	Richland, ND, USA	Soybean	0.5	+	30.64 ± 0.50

^a^ Each of the nematode samples used in the primer specificity tests were collected by the North Dakota State University Nematology Laboratory from different field locations in North Dakota, Minnesota, and Montana except for the *P. thornei* sample (sample ID: Pt), which was acquired from Oregon State University. Samples with the sample ID Hg50-1-4 are target species (*P. dakotaensis*) and the remaining samples in the table are non-target control species. The Hg51 nematode sample is another species of *Pratylenchus* newly identified in North Dakota (ND), USA, which is different from *P. dakotaensis*. ^b^ The crop that was planted during the growing season, in which the samples were collected. ^c^ For each of the control species used in the specificity tests, DNA was extracted from 2 individuals. For the target nematode species used in the specificity tests, DNA was extracted from 0.5, 1, 2, and 4 individuals. ^d^ The positive (+) and negative (−) symbols indicate that the target amplicon was detected or not detected, respectively, in conventional PCR assays with the designed primer set specific to *P. dakotaensis*. ^e^ The quantification cycle (Cq) value has been presented as the mean ± standard deviation of three technical replicates.

**Table 3:** Identification of *Pratylenchus* spp. collected from 20 soybean fields in North Dakota by species-specific polymerase chain reaction (PCR) assays ^a^.

Field ID	Crop	County Origin	Density in 100 cm^3^ of Soil ^b^	Conventional ^c^			RT ^d^	Species Identity
				PNEG-F1/D3B5	PsF7/PsR7	IC-ITS1 F/IC-ITS1R	IC-ITS1F/IC-ITS1R (Cq) ^e^	
50 RL 1	Soybean	Richland	21	−	+	−	N/A	*P. scribneri*
50 RL 2	Soybean	Richland	28	−	+	−	N/A	*P. scribneri*
50 RL 3 ^f^	Soybean	Richland	342	−	−	+	27.4 ± 0.1	*P. dakotaensis*
50 RL 4	Soybean	Richland	360	−	+	−	N/A	*P. scribneri*
C14L	Soybean	Dickey	140	−	+	−	N/A	*P. scribneri*
C3L	Soybean	Sargent	75	−	+	−	N/A	*P. scribneri*
HG50-0 ^g^	Soybean	Richland	360	−	−	+	27.7 ± 0.1	*P. dakotaensis*
Russ Field	Soybean	Wells	203	+	−	−	N/A	*P. neglectus*
SCN 188 E	Corn	Cass	45	+	−	−	N/A	*P. neglectus*
SCN 188 W	Corn	Cass	45	+	−	−	N/A	*P. neglectus*
SCN 207	Soybean	Cass	242	+	−	−	N/A	*P. neglectus*
SCN 215	Soybean	Grand Forks	120	+	−	−	N/A	*P. neglectus*
SCN 222	Soybean	Cass	100	+	−	−	N/A	*P. neglectus*
SCN 310	Soybean	Nelson	31	+	−	−	N/A	*P. neglectus*
SCN 311	Soybean	Nelson	30	+	−	−	N/A	*P. neglectus*
SCN 366	Dry bean	Grand Forks	60	+	−	−	N/A	*P. neglectus*
SCN 372	Soybean	Grand Forks	100	+	−	−	N/A	*P. neglectus*
SCN 388	Soybean	Grand Forks	25	+	−	−	N/A	*P. neglectus*
SCN 48	Soybean	Richland	15	−	+	−	N/A	*P. scribneri*
SCN 55	Corn	Richland	75	−	+	−	N/A	*P. scribneri*
SCN 7	Soybean	Cass	60	+	−	−	N/A	*P. neglectus*

^a^ For each sample, DNA was extracted from a single individual of *Pratylenchus* using the proteinase K method [31]. ^b^ Nematodes were extracted from 100 cm^3^ soil subsamples using centrifugal sugar floatation method, and the population density of *Pratylenchus* was determined under a microscope based on their morphological features [2,32]. ^c^ Each DNA sample was used in conventional PCR assays with 5 different species-specific primer sets to identify the species of *Pratylenchus* in each sample. The primer sets include PNEG-F1/D3B5 [22], PsF7/PsR7 [17], PTHO/D3B [22], PP5F/PP5R [23], and IC-ITS1F/IC-ITS1R (this study) that are specific to *P. neglectus*, *P. scribneri*, *P. thornei*, *P*. *penetrans*, and *P. dakotaensis*, respectively. However, none of the samples were positive for *P. thornei* and *P. penetrans*, thus results of those PCR assays were not included in this table. ^d^ Each sample was tested using real-time PCR assays with the IC-ITS1F/IC-ITS1R primer set, which is specific to *P. dakotaensis*. ^e^ Quantification cycle (Cq) value is presented as the mean ± standard deviation of three replicates. ^f^ 50 RL 3 is the field that is neighboring the field where P. dakotaensis was first detected. ^g^ HG50-0 is the soybean field where the P. dakotaensis was originally detected.

## Data Availability

All data generated or analyzed during this study are included in this article.
